# Core Values of Family Medicine in Europe: Current State and Challenges

**DOI:** 10.3389/fmed.2021.646353

**Published:** 2021-02-23

**Authors:** Eva Arvidsson, Igor Švab, Zalika Klemenc-Ketiš

**Affiliations:** ^1^Futurum, Jönköping, Sweden; ^2^School of Health and Welfare, Jönköping University, Jönköping, Sweden; ^3^Department of Family Medicine, Faculty of Medicine, University of Ljubljana, Ljubljana, Slovenia; ^4^Department of Family Medicine, Faculty of Medicine, University of Maribor, Maribor, Slovenia; ^5^Ljubljana Community Health Centre, Ljubljana, Slovenia

**Keywords:** value orientation, family practice, Europe, continuity of patient care, patient-centered care

## Abstract

**Background:** Values are deeply held views that act as guiding beliefs for individuals and organizations. They state what is important in a profession. The aims of this study were to determine whether European countries have already developed (or are developing) documents on core values in family medicine; to gather the lists of core values already developed in countries; and to gather the opinions of participants on what the core family values in their countries are.

**Methods:** This was a qualitative study. The questionnaire was distributed as an e-survey *via* email to present and former members of the European Society for Quality and Safety in Family Practice (EQuiP), and other family medicine experts in Europe. The questionnaire included six items concerning core values in family medicine in the respondent's country: the process of defining core values, present core values, the respondents' suggestions for core values, and current challenges of core values.

**Results:** Core values in family medicine were defined or in a process of being defined in several European countries. The most common core values already defined were the doctor-patient relationship, continuity, comprehensiveness and holistic care, community orientation, and professionalism. Some countries expressed the need for an update of the current core values' list. Most respondents felt the core values of their discipline were challenged in today's world. The main values challenged were continuity, patient-centered care/the doctor-patient relationship and comprehensive and holistic care, but also prioritization, equity, and community orientation and cooperation. These were challenged by digital health, workload/lack of family physicians, fragmentation of care, interdisciplinary care, and societal trends and commercial interests.

**Conclusion:** We managed to identify suggestions for core values of family medicine at the European level. There is a clear need to adopt a definition of a value and tailor the discussion and actions on the family medicine core values accordingly. There is also a need to identify the core values of family medicine in European countries. This could strengthen the profession, promote its development and research, improve education, and help European countries to advocate for the profession.

## Introduction

In recent years, the term “value-based practice” has emerged in discussions among professionals. It is one of the new approaches to supporting clinical decision-making where complex and sometimes conflicting values are in play (1). It includes values held by professionals and individual patients when making a decision (2). Integrating values in practice enables both patient values and evidence-based medicine to be used in decision-making (3).

There is some confusion about the definition of values in medicine. Namely, values can sometimes be proclaimed as principles, standards, or even competencies.

Values are deeply held views that act as guiding beliefs for individuals and organizations. They state what is important in a profession (4). Principles are the defining characteristics of the profession (5). They can be defined as a set of rules of the profession that stem from the core values of the profession. Standards state what is good or acceptable in a profession and can often change over time (4). A competence is one's ability to do the right thing at the right time, in the right way, in a specific complex professional context (6).

Values are important for the existence of the profession and for its vision. A profession needs to state clearly what it stands for. This defines the purpose of the profession and guides the governing of its affairs. While competencies, standards, and even principles can change, values usually remain the same over time (4).

In today's world morals and ethics are changing. Individual needs seem more important than ever, putting societal needs aside. Emerging technologies are changing the way we act, think, and interact. All this is affecting human values, and also medicine (and family medicine). Easy access to data is sometimes used to simplify primary care into small measurable units despite the fact that many of the most important things a GP does are not measurable (7). There is a decline in morale and motivation among family physicians and an increase in stress and burnout (8–12).

At times of great change, people and institutions often revisit their core values (4). This process has also been observed in family medicine in the last 40 years.

The debate about core values and principles started in the 1990s with Barbara Starfield's four pillars of primary care: first contact care, continuity, comprehensiveness, and coordination (13). After that, there were different attempts to define the core values (5). In 1998, Qureshi outlined 10 core principles of family medicine (14). Also, different suggestions emerged from European, Australian, and New Zealand general practice leadership (5, 15, 16). Several authors presented their ideas (4, 5, 17).

Little is known about the activities in European countries on family medicine core values. Also, there is still the need to define a universal set of core values at the European level (5). Therefore, we decided to conduct this study with the following aims: (1) to determine whether European countries have already developed (or are developing) documents on core values; (2) to gather the lists of core values already developed in countries; and (3) to gather the opinions of participants on what the core family values in their countries are.

## Materials and Methods

### Type of Study and Settings

This was a qualitative study. It was conducted in September/October 2020, in various European countries.

### Participants

The questionnaire was distributed to present and former members of the European Society for Quality and Safety in Family Practice (EQuiP), a European Organization consisting mainly of family doctors, whose aim is to promote quality improvement and patient safety in Europe. This group was selected since its members are interested and involved in developments in family medicine and were likely to be familiar with the situation in their countries. Additionally, other family medicine experts were invited when suggested by the EQuiP members.

### Questionnaire

The questionnaire included six questions or statements concerning core values in family medicine in the respondent's country. The first three questions concerned the process of defining core values and present core values, and the last three concerned the respondents' suggestions for core values and current challenges of core values, see Appendix 1.

### Data Collection

The questionnaire, together with information about the study, including that participation was voluntary, was distributed through email by a third party. The tool esMaker was used for the questionnaire and the data collection, which guaranteed the participants' anonymity.

The questionnaire was sent out to 79 individuals from 31 countries in September 2020. A reminder was sent out 3 weeks later, in October.

### Data Analysis

We performed a descriptive analysis of quantitative items. For analysis of open questions, the authors categorized the answers. For example, “continuous in time and on a personal level,” “from cradle to grave, relational continuity” and “continuous care” are all presented as “continuity.”

## Results

We obtained 23 replies from 14 countries. From Germany, the Netherlands, Slovenia and Sweden more than one person answered, and one person did not state his or her country (Table 1). In these cases, the respondents from the same country gave consistent answers and comments.

**Table 1:** Countries (number of respondents), current state of core values and links to website stating core values.

**Country (number of answers)**	**Current state of core values**	**Source**
Belgium (1)	Not yet defined	
Czech Republic (1)	Not yet defined	
Denmark (1)	Already defined	https://www.dsam.dk/pejlemaerker/
England (1)	Already defined	https://www.rcgp.org.uk/about-us/news/2017/july/rcgp-launches-strategic-plan.aspx
Germany (3)	Need for an update	https://www.degam.de/fachdefinition.html AND https://www.degam.de/what-we-do.html
Iceland (1)	Already defined	https://www.nfgp.org/flx/nfgp/core_values/ (The Icelandic version. “Grunngildi heimilislækna”)
Italy (1)	Not yet defined	
The Netherlands (3)	Already defined	https://toekomsthuisartsenzorg.nl/downloads/
Norway (1)	Definition in progress	
Poland (1)	Need for an update	http://www.cm-lancut.pl/asp/pl_start.asp?typ=14&menu=116&strona=1&sub=37
Romania (1)	Plans to start in the near future	
Serbia (1)	Not yet defined	
Slovenia (2)	Not yet defined	
Sweden (4)	Definition in progress	https://www.sfam.se/wp-content/uploads/2020/02/Vad-ar-allmanmedicin-sfam-dlf-presentation.pptx AND https://www.nfgp.org/flx/nfgp/core_values/
Unknown (1)		

### Process of Defining Core Values in Family Medicine

Respondents from four countries stated that core values in family medicine were already defined (Denmark, UK, Iceland, and the Netherlands), and respondents from five countries stated that core values had not yet been defined (Belgium, Czech Republic, Italy, Serbia, and Slovenia) (Table 1). In two of these countries, Belgium, Czech Republic, the competencies defined in the European Definition of General Practice/Family Medicine (18) were often used instead. The respondent from Norway stated that they were in the middle of the process of defining core values, and in Romania there were plans to start defining them in the near future.

The respondent from Poland wrote that core competencies had been defined many years ago, but had not been updated. In Germany, there was a similar situation, with a need for an update. The respondents from Sweden stated that the process of defining core values started many years ago and was still not finished, but their answers were somewhat contradictory. One person wrote that core values had already been defined and another said that the core values of the Nordic Federation of General Practitioners (16) were currently being translated into Swedish.

### Documents on Core Values in Family Medicine

In four countries where core values in family medicine had been defined (Denmark, England, Iceland, the Netherlands), they are described on the website of the family medicine organization so they can be accessed by everyone (Table 1). Some countries with non-updated core values also have digital access.

### Core Values, Already Defined in European Countries

Core values were already defined in six European countries (Denmark, England, Germany, Iceland, the Netherlands, and Sweden). All six had core values about the doctor-patient relationship and patient-centered care. Four of them reported core values concerning continuity, comprehensiveness and holistic care, and community orientation, coordination and cooperation.

Three countries had core values concerning excellence and professionalism and learning from practice.

Two countries had core values concerning teamwork, accessibility and avoiding over-diagnosis and overtreatment. Prioritization, equity and speaking up (e.g., against racism), health promotion, family orientation and leadership were each listed by one country (Table 2).

**Table 2:** Present and wanted core values as stated by the respondents.

**Core values as stated by the respondents**	**Number of countries where individual core value was already defined (answers from 6/14 countries)**
Patient-centered care/the doctor-patient relationship	6 (Denmark, England, Germany, Iceland, the Netherlands, and Sweden)
Continuity of care	4
Comprehensive and holistic care	4
Community orientation, coordination of care	4
Excellence, professionalism (including using best available evidence and participating in research, education and professional development) and learning from practice	3
Teamwork	2
Accessibility	2
Avoiding over-diagnosis and overtreatment	2
Prioritization	1
Equity	1
Health promotion	1
Family orientation	1
Leadership	1

### Suggestions for Core Values of Family Medicine

Respondents from 13 countries suggested core values for family medicine. The most commonly suggested core values were patient-centered care, and comprehensive and holistic care. These were followed by continuity of care, then excellence and professionalism (Table 2, Box 1).

Box 1 **Suggested core values of family medicine.**

**Table:** 

Patient-centered care Comprehensive care Holistic care Continuity of care Excellence and professionalism Accessibility	Community orientation Prioritization Teamwork Health promotion Family orientation and leadership Compassion	Usability Affordable care Universal coverage Advocacy Solidarity Patient empowerment

### Challenged Core Values

Most respondents agreed that traditional core values were challenged. Six answered “definitely,” 11 “very probably” or “probably,” two “possibly,” and two “probably not” or “definitely not.” All but two gave examples of current challenges of core values in family medicine, including the two who answered “probably not” or ”definitely not” to the question on whether core values are challenged.

The core value that most respondents thought was challenged was continuity. Patient-centered care and the doctor-patient relationship were also mentioned by several as being challenged. This was followed by comprehensive and holistic care, prioritization, equity, community orientation and cooperation, medicalization and over-diagnosis, and patient safety.

### Challenges to Core Values

The respondents recognized several challenges to core values of family medicine. The most common one was increased workload as a result of task-shifting from secondary to primary care, a decreasing number of GPs, and increasing demand from patients. Another challenge was teamwork and interdisciplinary care, seen by some as a threat to the holistic approach to patients.

It was recognized that societal trends and commercial interests could challenge patient-centered care and a continuous doctor-patient relationship.

Digital health was mentioned by several as another threat to the doctor-patient relationship, and especially the “digital-only companies” with doctors working without continuity or access to the patients' medical records.

Some respondents wrote that the GPs' role as a gate-keeper was changing and that patients “can go to any specialist without a referral or information from a GP.” Together with “patients' wish to get immediate access to care” this challenged core values such as continuity and the doctor-patient relationship. Fragmentation of care was considered a threat to comprehensive and holistic care by some respondents.

Equity was challenged in the opinion of some respondents as a challenge due to a problem with well-educated patients who might get more care despite lesser needs, and a growing number of people from different cultural backgrounds.

Other challenges included the funding systems, and the Covid-19 pandemic.

## Discussion

Our study showed that core values were defined or in a process of being defined in several European countries. The most common core values already defined were the doctor-patient relationship, continuity, comprehensiveness and holistic care, community orientation, and professionalism. Some countries expressed the need for an update. Most respondents felt the core values of the discipline were challenged in today's world. The main values challenged were continuity, patient-centered care/the doctor-patient relationship and comprehensive and holistic care but also prioritization, equity and community orientation and cooperation. These were challenged by digital health, workload/lack of family physicians, fragmentation of care, interdisciplinary care, and societal trends and commercial interests.

With our study, we identified almost the same core values as those proposed by Barbara Starfield in the 1990s. She identified first contact care, continuity, comprehensiveness, and coordination (13). It seems that the core values of the discipline remained stable through almost 30 years. This is an important finding as it may suggest that even if the family medicine practice changes (i.e., telemedicine, multimorbidity, more elderly people), the core values are still the same.

As evident from the responses obtained in our study, there is a misunderstanding about what core values mean and describe. Our respondents often described competencies or principles instead of values. For example, they stated that cooperation and health promotion were core values but they are more likely to be competencies. Some respondents even stated that they used the competencies described in the WONCA Europe document as core values. However, some of the examples/suggestions listed in Table 1 are good templates for further identifying the core values of the family medicine. For example, excellence and professionalism, prioritization (giving care to those who need it most), and leadership could very much be the real core values of family medicine.

Our study found that some values, principles and competencies are similar in most of the participating countries. They include patient-centered care, continuity of care, comprehensive care etc. (Table 2). However, some emerged only in some countries, such as teamwork, avoiding over-diagnosis and overtreatment, equity, family orientation, leadership etc. (Table 2). This might be a consequence of two issues. The first one is that the respondents had different understandings of what values are. So, it is possible that they listed also other items (such as competencies) not only core values. This could explain the different examples stated in Table 2. The other issue is the differences among European countries regarding health care systems, cultural background, working conditions etc. It has already been shown that such variables can affect the values, principles and competencies of a profession (19). Examples of this are the GPs' role as a gate-keeper, and teamwork where nurses and other professionals take over tasks that used to be performed by GPs. These findings point to a need for a discussion on the European level about whether common European core values of family medicine can be identified.

Our responders agreed that core values are challenged in today's world. The main challenges are associated with the changing societies. For example, the emerging fragmentation (20) of healthcare challenges continuity, and person-centered care. Continuity is also challenged by “modern” working hours. Technology and too much information challenge equity and patient safety. Medicalization also challenges patient safety. Furthermore, the covid-19 pandemic has challenged almost every core value, principle and competency (21).

An interesting finding was that interprofessional teamwork was sometimes considered a challenge to core values of continuity and the doctor-patient relationship. Interprofessional care for patients in primary care is nowadays seen as an advantage, especially for care of chronic patients (22, 23).

Challenges to core values are important since studies that show the benefits of primary care are based on the fact that it is characterized by continuity, person-centeredness, and comprehensiveness. If these long-lasting core values can no longer be maintained, there is a risk that primary care will not fulfill its important function in the health care system (24–26). It seems that some of the new developments in primary care should be carefully considered by professionals to assess possible positive and negative consequences.

Nevertheless, our findings indicate the need to reevaluate core values alongside changes in societies and human values, and determine whether it is necessary to redefine core values of family medicine to adapt to modern society and also how the ones that are still important can be maintained. It is important that young doctors are also active in this process so a shared vision of future primary care that is attractive to work in and has a strong position in the healthcare systems can be created. How to address the challenges to core values may, at least partly, evolve from the process of re-evaluating them.

Our study is to our knowledge the first study on the European level examining core values of family medicine. There are also some limitations. A difficulty with using a questionnaire in English is that persons with other mother tongues may misunderstand questions or express themselves in ways that might be interpreted ambiguously. An example is patient-centered care/the doctor-patient relationship, which can be considered two different entities, but were also described as one by some respondents. The patient-doctor relationship has many dimensions (27) which could have been further analyzed if we had conducted interviews instead of using questionnaires.

The response rate was low, 23/79, reflecting 14 different countries. Since not all European countries participated there could be a selection bias. Furthermore, even though the answers from the four countries where we got more than one answer were consistent, we cannot be sure whether the answers reflect the situation in a country or rather the opinion of the respondent. The low response rate could be the consequence of the lack of time of the physicians due to the corona pandemic, but it is also possible that they did not understand what core values are, and hence they chose not to answer.

Our study was qualitative, so we did not aim to perform any comparisons, for example according to different health care systems (28). However, in our opinion, our study gives a good insight into the problem at the European level and indicates the need for further action.

With our study, we managed to identify suggestions for core values of family medicine at the European level. However, there is a clear need to adopt a definition of a value and tailor the discussion and actions on the family medicine core values accordingly. The results of our study indicate a need to identify the core values of family medicine, which are still valid and important in a changing society, those that define our profession and are the same regardless of the diversity of European countries. This would strengthen the profession, promote its development and research, improve education, and help European countries to advocate for the profession.

Further studies are needed to become familiar with the field in individual European countries and to stimulate discussion on the matter at the European level. We suggest a series of qualitative studies, and possibly in-depth interviews, among experts in family medicine, practicing family physicians, and also patients to obtain a deeper insight into the topic. The research should also focus on possible differences among core values in different countries according to health care system differences.

## Data Availability Statement

The original contributions presented in the study are included in the article/supplementary material, further inquiries can be directed to the corresponding author.

## Author Contributions

EA and ZK-K conceived the study, interpreted the data, and wrote the first draft of the manuscript. EA, ZK-K, and IŠ developed the methodology of the study. EA collected the data. IŠ revised the first draft of the manuscript. All authors read and approved the final version of the manuscript.

## Conflict of Interest

The authors declare that the research was conducted in the absence of any commercial or financial relationships that could be construed as a potential conflict of interest.

## Appendix 1: Questionnaire

Please, state the country you are based in:

1. Have you started the process of defining core values in family medicine in your country?
(Yes, we are currently in the middle of the process./Already finished/No, but we plan to start it near future/No/Other)
If other, please describe
2. Have the family medicine organizations in your country already developed documents on core values in family medicine?
(Yes/No)
If yes, please, state the name of the document, and if possible, a link.
3. If you have core values in family medicine defined within your country, please, list them including the related source (organization, document), if possible.
4. What do you think core values in family medicine should be? Please, list them.
5. you think that traditional core values are challenged now in this changing world due to IT in our practices, fragmentation etc.?
(Definitely, Very Probably, Probably, Possibly, Probably Not, Definitely Not)
Comments:
6. Do you have examples of current challenges of core values in family medicine from your country?
(Yes/No)
If yes, please describe:
